# Simulating Longitudinal Brain MRIs with Known Volume Changes and Realistic Variations in Image Intensity

**DOI:** 10.3389/fnins.2017.00132

**Published:** 2017-03-22

**Authors:** Bishesh Khanal, Nicholas Ayache, Xavier Pennec

**Affiliations:** Asclepios, INRIA Sophia Antipolis MediterranéSophia Antipolis, France

**Keywords:** neurodegeneration, biophysical modeling, biomechanical simulation, simulated database, synthetic images, synthetic longitudinal MRIs

## Abstract

This paper presents a simulator tool that can simulate large databases of visually realistic longitudinal MRIs with known volume changes. The simulator is based on a previously proposed biophysical model of brain deformation due to atrophy in AD. In this work, we propose a novel way of reproducing realistic intensity variation in longitudinal brain MRIs, which is inspired by an approach used for the generation of synthetic cardiac sequence images. This approach combines a deformation field obtained from the biophysical model with a deformation field obtained by a non-rigid registration of two images. The combined deformation field is then used to simulate a new image with specified atrophy from the first image, but with the intensity characteristics of the second image. This allows to generate the realistic variations present in real longitudinal time-series of images, such as the independence of noise between two acquisitions and the potential presence of variable acquisition artifacts. Various options available in the simulator software are briefly explained in this paper. In addition, the software is released as an open-source repository. The availability of the software allows researchers to produce tailored databases of images with ground truth volume changes; we believe this will help developing more robust brain morphometry tools. Additionally, we believe that the scientific community can also use the software to further experiment with the proposed model, and add more complex models of brain deformation and atrophy generation.

## 1. Introduction

Structural Magnetic Resonance Imaging (MRI) has been widely used for *in vivo* observation of morphological changes over time in human brain. Atrophy or tissue volume loss measure from structural MRI is an established biomarker for neurodegeneration (Frisoni et al., 2010). There is a large number of brain morphometry algorithms developed in the literature which estimate global or local atrophy from structural MRIs (Wright et al., 1995; Freeborough and Fox, 1997; Ashburner and Friston, 2000; Smith et al., 2002; Hua et al., 2008). Volume/atrophy measurements obtained from such algorithms have been used to test various clinical hypotheses about neurodegenerative diseases (Wright et al., 1995; Sepulcre et al., 2006; Koch et al., 2016). Similarly, comparison of different neurodegenerative diseases have also been performed based on these measurements (Rosen et al., 2002; Whitwell and Jack, 2005). Since atrophy estimation is an inverse problem, the estimation algorithms require a model with certain parameters. The results obtained from such algorithms depend on model assumptions and the parameters used. Often, these assumptions are implicit and cannot be directly linked to the biophysical process of neurodegeneration. For instance, tensor based morphometry (TBM) encodes local volume changes by computing Jacobian determinants of the deformation field obtained from non-linear registration of longitudinal MRIs (Ashburner and Ridgway, 2015). Such methods contain model biases because TBM results depend on the choices of regularization used during the registration of images (Ashburner, 2013). Likewise, edge-based methods such as BSI, SIENA etc. are sensitive to unmatched image contrasts between scans, poor signal-to-noise ratio, partial volume effects, segmentation errors etc. (Preboske et al., 2006; Prados et al., 2015). Estimating and correcting the bias present in such morphometry tools is important, especially for clinical applications.

In addition to tracking volumetric changes in specific brain structures, longitudinal imaging data can also be used to study the temporal inter-relationship of atrophy in different structures. For instance, Carmichael et al. (2013) studied the groupings of 34 cortical regions and hippocampi from the per-individual rates of atrophy estimates in these regions. In Fonteijn et al. (2012), authors defined AD progression as a series of discrete events. Along with other clinical events, the timings of atrophy in various brain structures were included in a set of discrete events. Without any prior to their ordering, the model finds the most probable order for these events from the data itself. They used Bayesian statistical algorithms for fitting the event-based disease progression model. The objective of these studies were to understand how different regions of brain evolve during the neurodegeneration.

In this context of increasing use of the atrophy measurements from longitudinal MRIs in testing or discovering clinically relevant hypotheses, it is important to study the bias and variability of the atrophy estimation algorithms. The actual volume changes in real longitudinal MRIs are not known. Thus, the evaluation and validation of atrophy estimation algorithms require generating images with known volume changes, called ground truth images.

A number of atrophy simulators have been proposed in the literature to produce ground truth MRIs (Smith et al., 2003; Camara et al., 2006; Karaçali and Davatzikos, 2006; Pieperhoff et al., 2008; Sharma et al., 2010; Modat et al., 2014; Radua et al., 2014; Khanal et al., 2016a). Most of these simulators use a model that attempts to produce a deformation field with the specified volume changes in the input brain MRI. To produce realistic scenarios of noise and acquisition artifacts, some of these simulators also use a model to produce noise and artifacts in the simulated image.

Such simulators have been used for the validation of registration or segmentation based atrophy estimation algorithms (Camara et al., 2008; Pieperhoff et al., 2008; Sharma et al., 2010), to estimate the bias in such algorithms, and also to estimate uncertainty in the measured atrophy (Sharma et al., 2013). These studies have estimated the bias by simulating simple atrophy patterns in a small number of brain regions or uniform diffused global atrophies. However, real case scenarios could have a much more complex atrophy distribution occurring in many brain structures at the same time.

Noise and imaging artifacts have an important impact on the results obtained from atrophy estimation algorithms (Camara et al., 2008; Pieperhoff et al., 2008; Sharma et al., 2010). Thus, proper evaluation of atrophy estimation algorithms by using simulated ground truth images requires simulation of realistic variation in noise and intensity too. All the previous atrophy simulators have warped the input baseline image with the deformation field obtained from a model of brain deformation. Then, extra noise and artifacts are added on this warped image by using another artificial model. The intensity noise in structural MRIs has been shown to be governed by a Rician distribution where the noise is Gaussian in *k*-space (Gudbjartsson and Patz, 1995). Thus, the Rician noise can be added in the simulated images as follows:

- Use two independent random variables following zero-mean Gaussian distribution to compute the real and imaginary parts of a complex number at each voxel.
- Considering the original intensity to be a complex number with zero imaginary part, add the real and imaginary components obtained above and take the magnitude of the resulting complex signal.

For example, Sled et al. (1998) used this approach to add noise in simulated MRIs that were used for the validation of intensity bias correction scheme they presented. Using the same approach, Camara et al. (2008) added noise to the simulated ground truth images with atrophy.

In addition to the Rician noise described above, other noise, and artifacts are also present in MRIs (Simmons et al., 1994). Some of the artifact sources that have been shown to affect the measurements of atrophy estimation algorithms (Camara-Rey et al., 2006; Pieperhoff et al., 2008; Sharma et al., 2010) are:

- Bias field inhomogeneity arising due to poor radio frequency (RF) coil uniformity.
- Geometrical distortions that are present due to the errors in gradient field strength and non-linearity of gradient fields in the MR scanner (Langlois et al., 1999).
- Interpolation of intensities during various pre-processing steps of TBM based analysis framework (e.g., resampling of the images into a common template space).

Many other acquisition artifacts may not be simulated because we do not have faithful models. Inability to produce realistic intensity variation and noise in simulated longitudinal images is one of the key limitations in the state-of-the-art atrophy simulators, including our previous work (Khanal et al., 2016a). In this work, we propose a simple but elegant solution to remove the limitation of previous atrophy simulators. First, our biophysical model of brain deformation (Khanal et al., 2016a) is used to obtain a dense deformation field with specified volume changes. Then, to obtain realistic intensity variations, intensities in the simulated images are resampled from baseline repeat scans of the same patient. Although the method is very simple and straightforward, this allows simulating longitudinal images with variation in intensity and noise taken from real scans themselves without explicitly specifying any noise or artifact models. To the best of our knowledge, this idea was not presented before in the literature. When the repeat scans are not available, we use an approach introduced by Prakosa et al. (2013) where the authors simulate visually realistic time series of cardiac images. Intensity variation in the simulated images of a patient is obtained by resampling the intensities from the repeat scans if available, otherwise from the real images of the same patient taken at different times.

**Figure 1:**
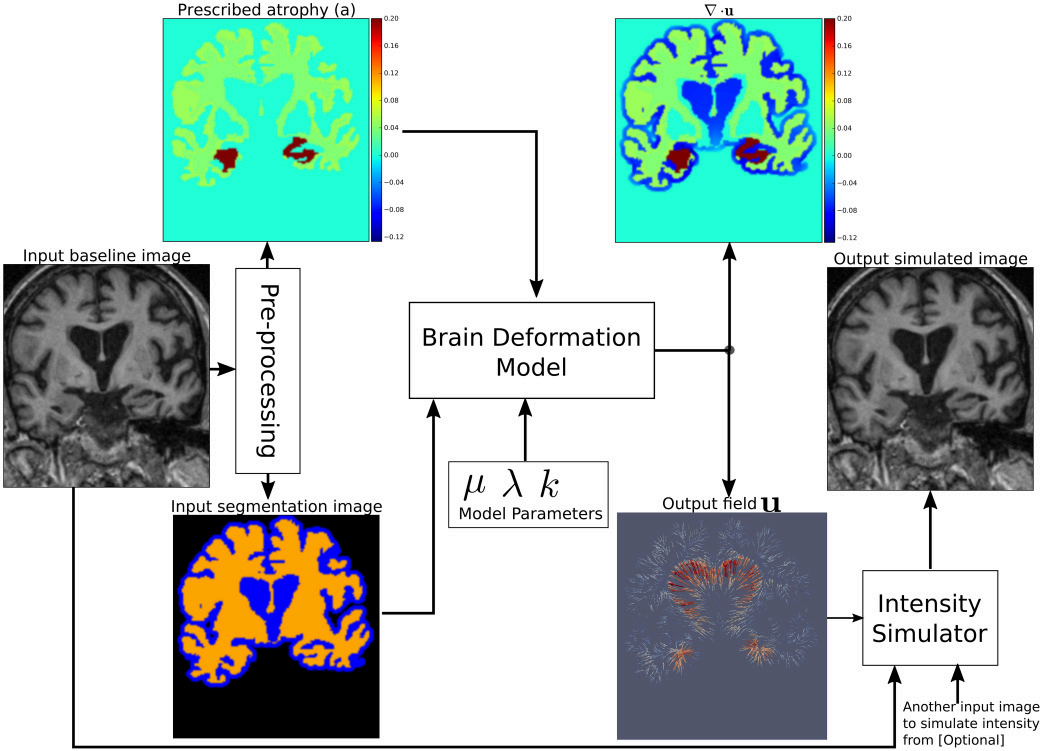
**Pipeline to simulate synthetic images using Simul@trophy**. Starting from a real baseline image of a subject, synthetic images with known volume changes can be generated. These synthetic images can follow intensity characteristics of either the input baseline or other images of the same subject. Pre-processing is required to generate an atrophy map and a segmentation image, which are fed as inputs to the brain deformation model. For a given set of parameters, the model computes a velocity field whose divergence is equal to the prescribed atrophy map at each voxel of the regions selected by using the segmentation image. Intensity simulator uses the output field to produce synthetic image whose intensity is resampled either from the input real baseline or from any other image as desired.

Figure 1 shows a diagram of the complete framework. To implement this framework, we have developed an open-source atrophy simulator software called Simul@trophy^1^. To our knowledge, Simul@trophy is the first atrophy simulator to be made open-source. Simul@trophy uses the biophysical model presented in Khanal et al. (2016a) but introduces a new numerical scheme to compute divergence, which removes the numerical inconsistency presented in the previous work. This is further explained in detail in Section 4.2.

Section 2 explains all the blocks of the framework shown in Figure 1. Starting from a small set of real scans, we show how longitudinal images with different atrophy patterns and realistic intensity variations can be simulated. Section 3 shows some simulation results using Simul@trophy, and also illustrates some potential applications of the simulator. In Section 4, we present some example simulations to illustrate some of the important points to consider when using Simul@trophy for different applications, such as evaluation of atrophy estimation algorithms, validation of data-driven disease progression models, training of brain morphometry algorithms based on machine learning etc.

## 2. Simulating realistic longitudinal images with atrophy/growth

We use the biophysical model presented in Khanal et al. (2014, 2016a) to generate dense deformation field with specified complex patterns of volume changes. This deformation field is then used to generate realistic synthetic longitudinal images with intensity variation, noise, and artifacts, just like in real longitudinal images. The major components of the simulation framework, as seen in Figure 1, are: (i) Pre-processing, (ii) Brain deformation model, (iii) Realistic intensity simulator.

### 2.1. Pre-processing to generate a segmentation image and atrophy maps

A pre-processing step takes a real scan of a patient as an input baseline image, and generates the required inputs of the brain deformation model: a segmentation image and a specified atrophy map.

#### 2.1.1. Segmentation image

There are three labels in the segmentation image used by Simul@trophy (Figure 1):

- Label0: regions where no deformation should be prescribed,
- Label1: regions where the deformation model is allowed to adapt volume changes as required,
- Label2: regions where certain volume changes are prescribed (the values of volume changes are provided with an input atrophy map).

Pre-processing usually starts with a brain extraction that excludes the skull and outside regions (also called skull stripping). Skull stripping is followed by a segmentation such that each voxel of the input image could be assigned to one of the three labels. For example, a typical pre-processing step that includes a segmentation of brain parenchyma and CSF would produce a segmentation image with the following labels:

- Label0: Skull and outside regions of the input image,
- Label1: CSF regions,
- Label2: Gray and white matter regions.

#### 2.1.2. Atrophy map

An atrophy map is a scalar image with desired values of volume changes in Label1 regions of the segmentation image, and zeros in all the other regions. It is defined at each voxel as follows:

$$a=\frac{V_{0}-V_{1}}{V_{0}},$$

where *V*_0_ and *V*_1_ are the volumes of the material lying in a voxel at time *t*_0_ and *t*_1_, respectively. Thus, regions with volume loss have positive values of *a* while the regions with volume expansion have negative values of *a*. An example atrophy map is shown in Figure 1. In this work, we illustrate example simulations where two kinds of pre-processing steps were used to generate the atrophy maps:

**Segmentation based atrophy map**

The user can set uniform values of atrophy in regions of interests (ROIs) of the brain. In this case, one must first perform a segmentation of all ROIs in which a non-zero value of atrophy is desired. Then, it is straightforward to create a scalar image having intensity values taken from a table, which contains the labels of ROIs and the corresponding desired atrophy values.

**Registration based atrophy map**

The results of longitudinal non-rigid registration can be used to estimate local volume changes, for instance by computing Jacobian determinants of the displacement fields or by computing the divergence of the stationary velocity fields obtained from the registration. These local volume changes obtained from the registration based methods are usually smoothly varying in space and can be used to prescribe either:

- smoothly varying atrophy maps,
- or atrophy maps uniform in ROIs obtained by averaging, in each ROIs, the atrophy obtained above.

**Figure 2:**
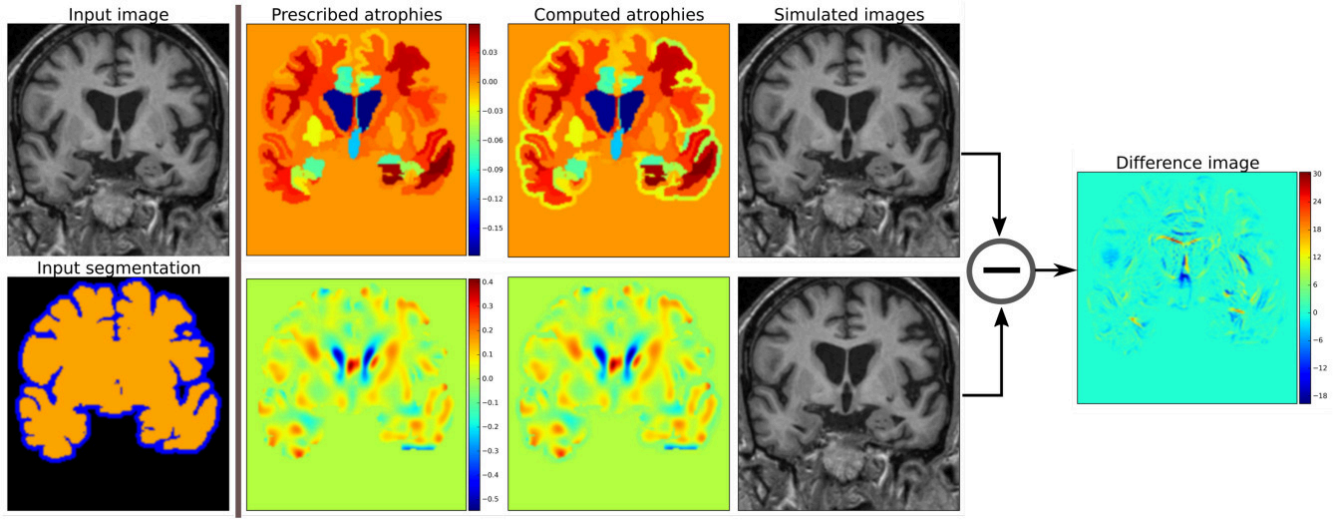
**Examples of two different kinds of atrophy maps**. The first row prescribes an atrophy map that is uniform in different regions of the brain, while the second row prescribes a smoothly varying atrophy. Both of these atrophy maps have same average values in each ROIs. The example also shows that we can prescribe volume changes in ventricles, if desired, by adapting the input segmentation map accordingly. The simulated images, as shown, are different although they have same mean regional atrophy values. The prescribed atrophy maps and the corresponding computed atrophy maps have different values of atrophy in the regions with sulcal CSF because it is part of Label1 (blue color in the segmentation map) where the volume is allowed to freely change.

Figure 2 shows two such atrophy maps with very different patterns, but having the same average regional volume changes.

### 2.2. A biophysical model of brain deformation with prescribed volume changes

Simul@trophy uses the biomechanics based model of brain deformation detailed in Khanal et al. (2016a). The model abstracts the phenomenon that evolves during several months or years in the brain at a macroscopic scale. It is based on the assumption that atrophy creates an internal stress which results in the deformation minimizing a strain energy. In other words, the brain parenchyma deforms with the prescribed atrophy by minimizing the strain energy. The strain energy corresponding to the prescribed atrophy at each time step is completely released when starting the next time step, which leads to a creep flow model.

For a given segmentation image, the model yields a deformation field with the prescribed atrophy at each voxel of Label2 regions (e.g., brain parenchyma). Label1 regions (e.g., the CSF) will correspondingly adapt its volume to globally compensate for the prescribed volume changes in the Label2 regions. For a single time-step, the displacement field **u** is obtained by solving the system of Equation (1), where Dirichlet boundary conditions of zero deformation are prescribed in Label0 regions.

(1) $$\begin{array}{l} \begin{array}{l} \text{Regions with: }\text{L}\text{a}\text{b}\text{e}\text{l}0\\ u=0\\ \text{Dirichlet boundary conditions} \end{array}\}\;\begin{array}{l} \text{L}\text{a}\text{b}\text{e}\text{l}1\\ \mu \Delta u-\nabla p\;=\;0\\ \nabla \cdot u+kp=0 \end{array}\}\\ \begin{array}{l} \text{L}\text{a}\text{b}\text{e}\text{l}2\\ \mu \Delta u-\nabla p=\;(\mu +\lambda )\nabla a\\ \nabla \cdot u=-a \end{array}\} \end{array}$$

The system of Equation (1) shows that the incompressibility constraint is relaxed in Label1 regions, while it is strictly satisfied in Label2 regions.

The prescribed atrophy map *a* in the constraint ∇ · **u** = −*a* is the amount of atrophy in a small time step Δ*t* such that the displacement field **u** and its gradient are small enough to make the following approximation: ∇ · **u** = −*a* ≈ *J* − 1, where *J* is the Jacobian determinant (Khanal et al., 2016a). Jacobian determinant measures the relative volume of a warped voxel, *V*_1_/*V*_0_.

The impact of the choice of different values for the model parameters μ, λ, and *k* are detailed in Khanal et al. (2016a). For the same prescribed volume changes, we can obtain different deformation fields by varying these model parameters. In this work, we focus on generating ground truth images with known volume changes and not necessarily generating the exact evolution of the AD patients. Hence, we set the model parameters as follows unless specified otherwise: μ = 1 kPa, λ = 0 kPa, *k* = 1 kPa^−1^.

Once the field **u** with the prescribed volume changes is obtained from the model as described above by using an input baseline image *I*_*b*_, we can simulate a synthetic follow-up image *I*_*s*_ as follows:

- Let **y** = Φ_sim_(**x**) = **u** + **x** describe a mapping of a point **x** in physical space to another point **y** by applying the transformation corresponding to the dense deformation field Φ_sim_, or the displacement field **u**.
- Let Φ_sim_⋆*I*_*b*_ describe an action of the diffeomorphism Φ_sim_ on the image *I*_*b*_. Thus, the new synthetic image *I*_*s*_, obtained by warping *I*_*b*_ with the deformation field Φ_sim_ is given by:
  $$I_{s}=\Phi_{\text{sim}}⋆I_{b}=I_{b}\circ \Phi_{\text{sim}}^{-1}.$$

Figure 2 shows two simulated images from the same input baseline image but with two different atrophy patterns.

### 2.3. Adding realistic intensity variation to synthetic longitudinal MRIs

In realistic scenarios, longitudinal MRIs are taken at multiple scan sessions often with slightly different acquisition parameters or even with different scanners. For generating more realistic synthetic longitudinal MRIs, variations in intensity, and noise present in real longitudinal MRIs must also be simulated. If multiple repeat scans of a subject are available, we can use them to simulate such variations in synthetic longitudinal sequences. Assuming that all the available scans of the subject are already aligned using affine registration, this section explains the proposed method of adding realistic variations in the intensity characteristics.

Starting from an input baseline image *I*_*b*_0__ of a subject, the previous sections explained how we can obtain a deformation field Φ_sim_ from the brain deformation model, and use it to simulate a follow-up image

$$I_{s_{0}}=\Phi_{\text{sim}}⋆I_{b_{0}}.$$

*I*_*s*_0__ has the same intensity characteristics as *I*_*b*_0__, and the intensity noise in *I*_*s*_0__ is strongly correlated to the noise present in *I*_*b*_0__.

If *I*_*b*_1__ is another scan of the same subject taken on the same day, we can obtain a new simulated image by resampling the intensity from *I*_*b*_1__, but still using the same Φ_sim_:

$$I_{s_{1}}=\Phi_{\text{sim}}⋆I_{b_{1}}$$

The realistic variation of intensity and artifacts present between the two real scans *I*_*b*_0__ and *I*_*b*_1__ are now also present between the real baseline image *I*_*b*_0__ and the simulated follow-up image *I*_*s*_1__.

The above approach assumes that the brain has not undergone any morphological changes between the scan sessions of the two real images. If the scan time-points of the two images are too far apart to have this assumption valid, we can no longer directly apply Φ_sim_ to the second image. Let *I*_*r*_ be another real scan of the patient taken at a time later than that of the baseline image *I*_*b*_0__. There might be some morphological changes (e.g., atrophy) in *I*_*r*_ compared to *I*_*b*_0__.

To simulate a new synthetic image with the same atrophy as that of *I*_*s*_0__ but with the intensity resampled from *I*_*r*_, we must first perform a non-rigid registration between *I*_*r*_ and *I*_*b*_0__. If Φ_reg_ is the deformation field obtained from the non-rigid registration between *I*_*r*_ and *I*_*b*_0__, it can be used to get an image Φ_reg_⋆*I*_*r*_ which is aligned to *I*_*b*_0__. In the ideal case, Φ_reg_⋆*I*_*r*_ and *I*_*b*_0__ are perfectly aligned with the only differences lying in the intensity characteristics and the noise.

We can now compose the deformation fields Φ_sim_ and Φ_reg_ to generate a new synthetic image as follows:

$$I_{s_{2}}=\left(\Phi_{\text{sim}}\circ \Phi_{\text{reg}}\right)⋆I_{r}.$$

**Figure 3:**
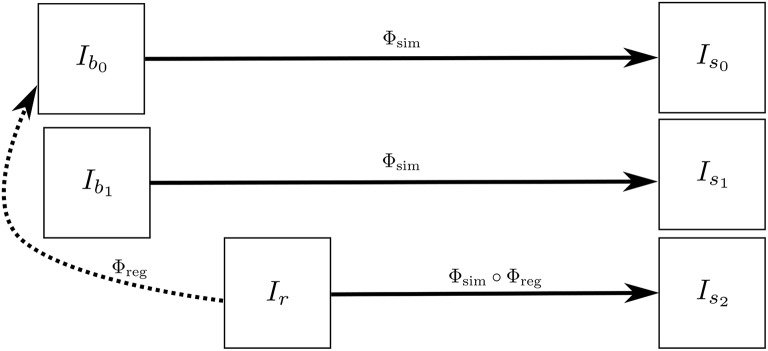
***I*_*b*_0__ and *I*_*b*_1__ are the repeat scans of a subject taken within a short period of time during which there is no morphological changes in the brain of the subject**. *I*_*r*_ is taken at a later time when the brain could have undergone some morphological changes. The deformation field Φ_reg_ is obtained by registering *I*_*r*_ to *I*_*b*_0__, while Φ_sim_ is obtained from the brain deformation model using *I*_*b*_0__ as the input image. The three simulated images *I*_*s*_0__, *I*_*s*_1__, and *I*_*s*_1__ are all same time-point images but have different intensities that come from *I*_*b*_0__, *I*_*b*_1__, and *I*_*r*_, respectively.

*I*_*s*_2__ has the same atrophy as that of *I*_*s*_0__ but with the intensity characteristics of *I*_*r*_. Figure 3 illustrates how we obtain *I*_*s*_0__, *I*_*s*_1__, and *I*_*s*_2__. These three simulated images have the volume changes as encoded by Φ_sim_, but have intensity characteristics coming from three different real images of the same patient.

**Figure 4:**
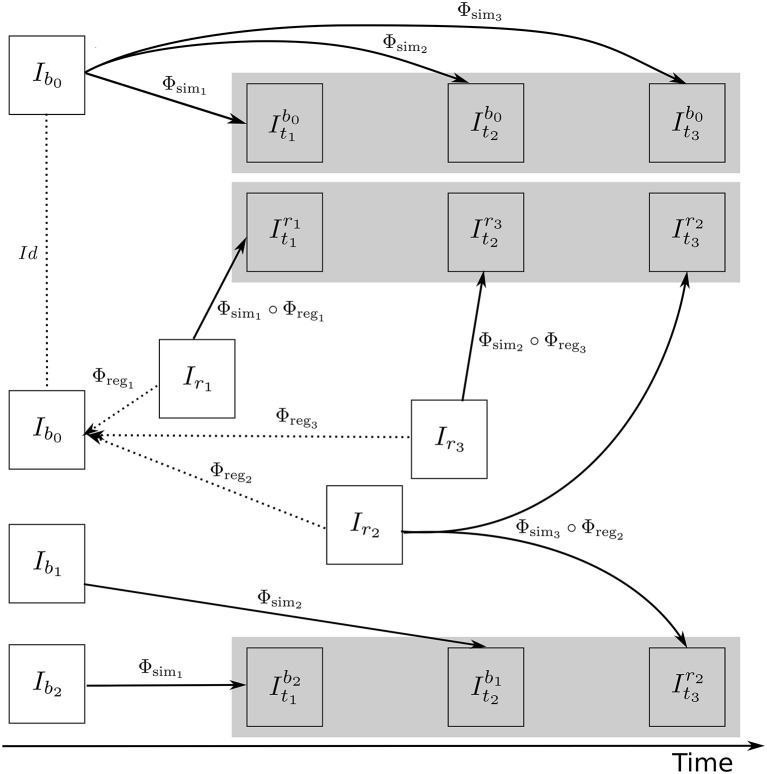
**A general approach to simulate ground truth synthetic longitudinal images with realistic intensity variations; simulated images are shown within the shaded regions**. The deformation fields with a prescribed atrophy for three time-points (Φ_sim_1__, Φ_sim_2__, and Φ_sim_3__) are obtained from the biophysical model using *I*_*b*_0__ as the input baseline image. Several different sets of longitudinal images can then be simulated by resampling intensities from different combinations of available real images. The topmost shaded region shows a longitudinal sequence with no realistic intensity variations where the synthetic images are all resampled from *I*_*b*_0__. The remaining two shaded regions have longitudinal sequences with realistic intensity variations where the simulated images are resampled from other available images of the same subject. In the ideal case, the three sets of longitudinal sequences have exactly the same morphological changes but with different variations in intensity characteristics.

Figure 4 illustrates how the approach described in this section can be used to generate multiple sets of longitudinal simulated sequences having identical morphological evolution but different variations of intensities. The three shaded regions in Figure 4 are the sets of longitudinal sequences with identical volume changes but with different variations of intensities.

## 3. Simulation examples with Simul@trophy

This section presents simulation examples of synthetic longitudinal MRIs with prescribed atrophy patterns and realistic intensity variations^2^. The real input MRIs used for the simulations presented in this section come from the database made available by Hadj-Hamou et al. (2016). The images had already undergone the Pre-Processing and Position Correction steps of the Longitudinal Log-Demons Framework (LLDF) detailed in Hadj-Hamou et al. (2016). Starting from the publicly available OASIS dataset (Marcus et al., 2010), the images in the database had undergone intensity inhomogeneity correction using ANTs–N4BiasFieldCorrection (Avants et al., 2011), and had been transported to a common space using affine registration with FSL–FLIRT (Jenkinson and Smith, 2001).

Since all the simulated images must undergo interpolation of intensities, numerical scheme used in the interpolation will have an impact on the intensity characteristics of the simulated images. In all the simulation examples that follows, intensities were resampled using B-spline interpolation of order 3.

**Figure 5:**
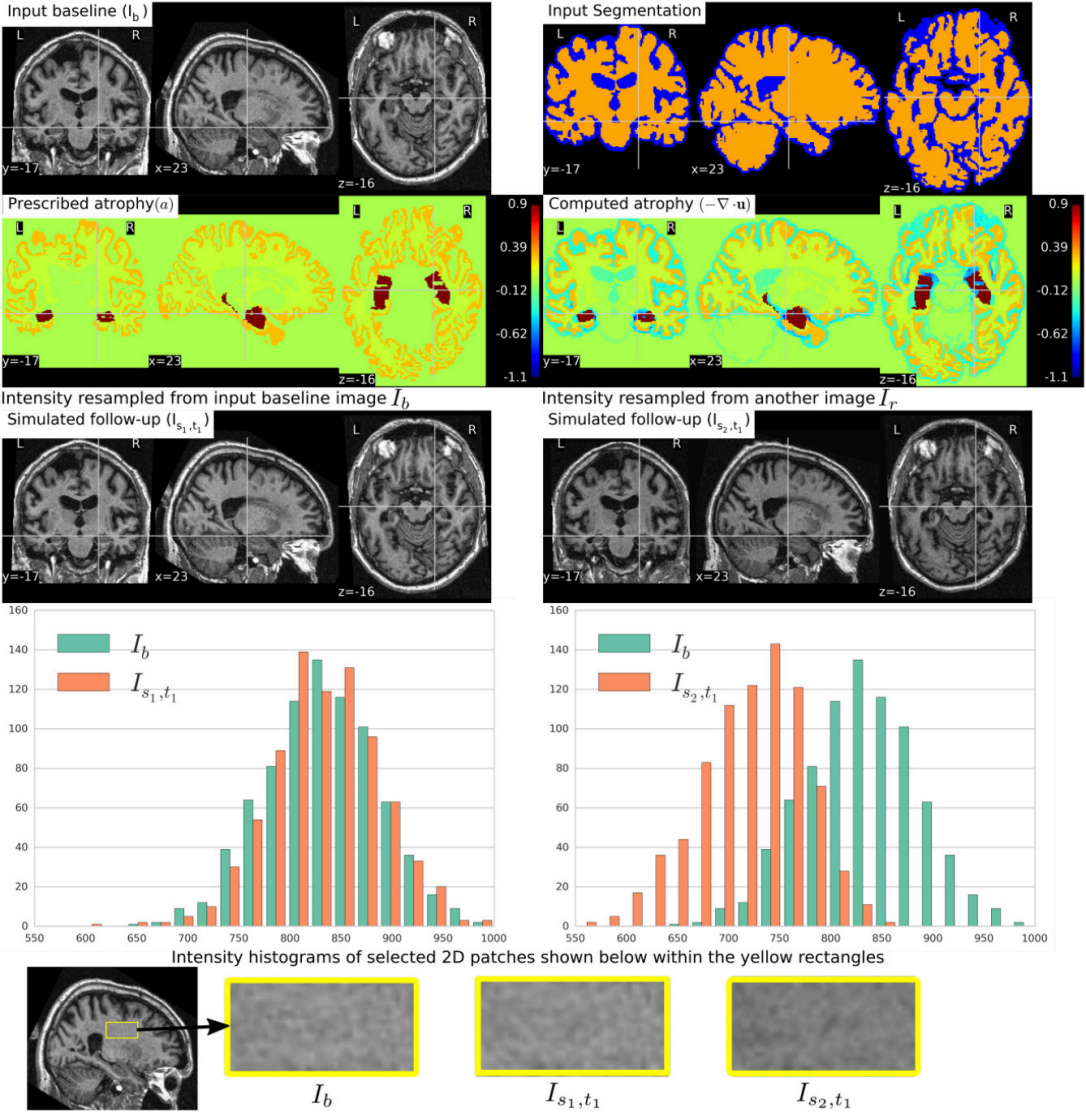
**Two simulated images are shown on the third row where the image on the left is resampled from the input baseline image *I*_*b*_, and the image on the right is resampled from another image *I*_*r*_ of the same subject**. Both *I*_*b*_ and *I*_*r*_ had already been corrected for the bias field intensity inhomogeneity. The intensity histograms shown are of a selected ROI (shown on the last row) where there is no significant morphological changes between the images. From the histograms we can see that the simulated image *I*_*s*_2_, *t*_1__ has a different intensity characteristics than *I*_*b*_, while the simulated image *I*_*s*_1_, *t*_1__ has intensity characteristics that closely matches to that of *I*_*b*_.

Figure 5 shows a simulation example where uniform atrophy patterns are prescribed in the hippocampi, the gray matter (GM), and the white matter (WM) regions. The ventricles and sulcal CSF regions are allowed to expand as required to compensate for the volume loss in the brain parenchyma. The figure shows two simulated images whose intensities are resampled from two different images: (i) the input baseline image *I*_*b*_, (ii) another follow-up image of the same subject, *I*_*r*_. The figure also shows intensity histograms of these two simulated images for a selected ROI. The selected ROI is a 2D WM region where the simulated images do not have a distinct morphological changes from *I*_*b*_. Thus, the differences in the intensity histograms of *I*_*b*_ and the simulated images for this ROI is mostly due to the variation in intensity characteristics of the different images. We can see from the figure that the intensity characteristics of the simulated image resampled from *I*_*b*_ closely matches the intensity characteristics of *I*_*b*_. And resampling the intensity from a different image *I*_*r*_ of the same subject allows simulating realistic variation of intensities.

To simulate multiple time-point images, the following approach can be used:

- Get **u**_0_ by solving the system of Equation (1) using the initial atrophy map *a*_0_ and the initial segmentation image *L*_0_ as input.
- For each time step *t* = 1 to *n*:
  - Warp *a*_*t*−1_ and *L*_0_ using **u**_*t*−1_ ∘ **u**_*t*−2_… ∘ **u**_0_ to get *a*_*t*_ and *L*_*t*_, respectively.
  - Solve for **u**_*t*_ using *a*_*t*_ and *L*_*t*_ as input.

Once all the deformation fields Φ_*s*_*i*__ corresponding to **u**_*i*_ for *i* = 0, 1, …, *n* are obtained, these deformation fields can be used as shown in Figure 4 to simulate different sequences of longitudinal images. As time step gets larger, the segmentation map is warped with an increasingly bigger displacement field using nearest neighbor interpolation, which could result in numerical instabilities. As the atrophy map is also warped at each time step, the global atrophy rate prescribed in the beginning is not necessarily preserved during the intermediate time-steps.

**Figure 6:**
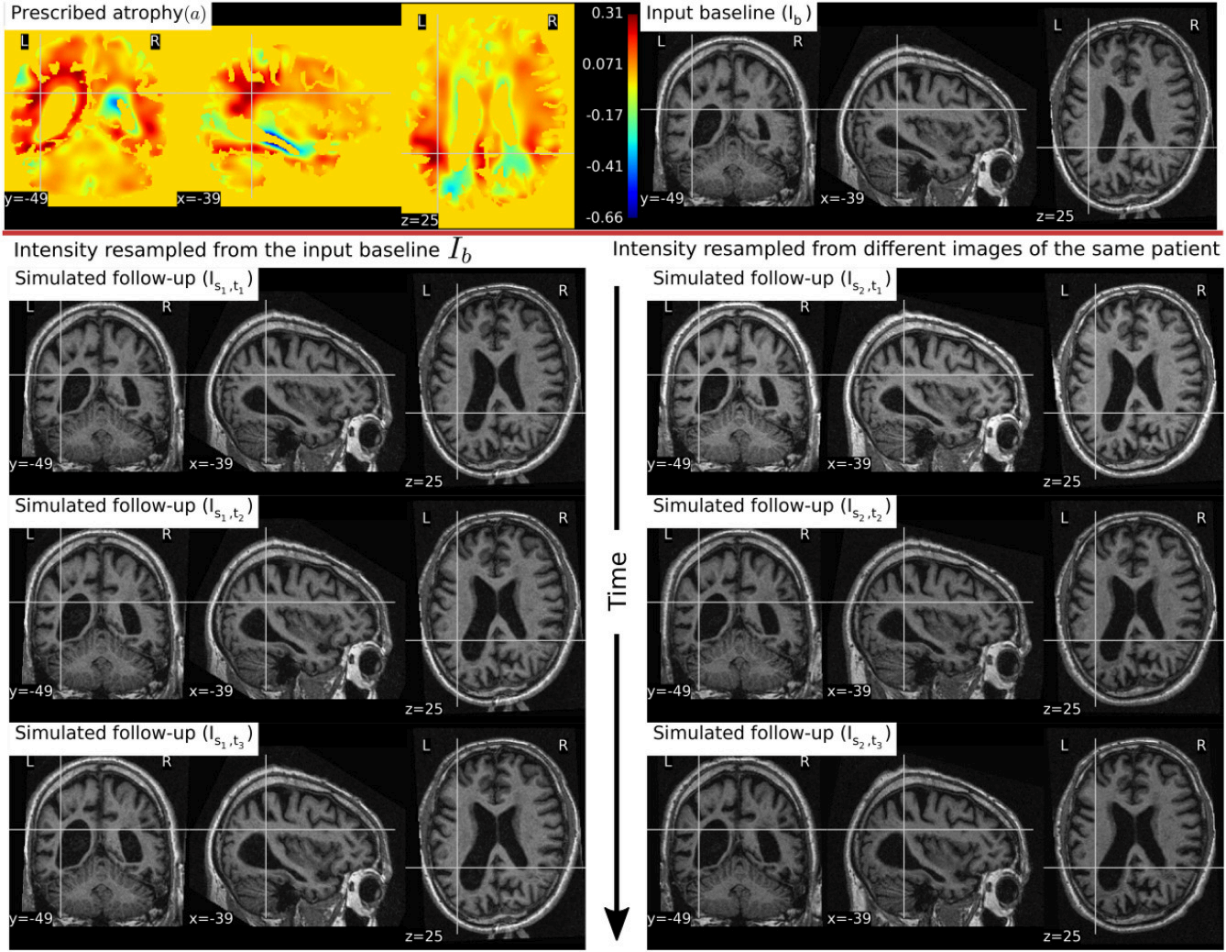
**Two sets of synthetic longitudinal images are shown which are simulated by prescribing a smoothly varying atrophy pattern**. The first row shows the input prescribed atrophy and the input baseline image *I*_*b*_ of a subject, while the remaining rows show the two sequences. The sequence shown on the left have simulated images that are all resampled from *I*_*b*_. On the right, each simulated image is resampled from real MRIs of the same subject but taken at different times (at 0.68, 1.77, and 3.3 years after the baseline scan respectively). As shown by the intensity histograms of Figure 7, the longitudinal synthetic images on the right have more realistic intensity variations than the one left.

**Figure 7:**
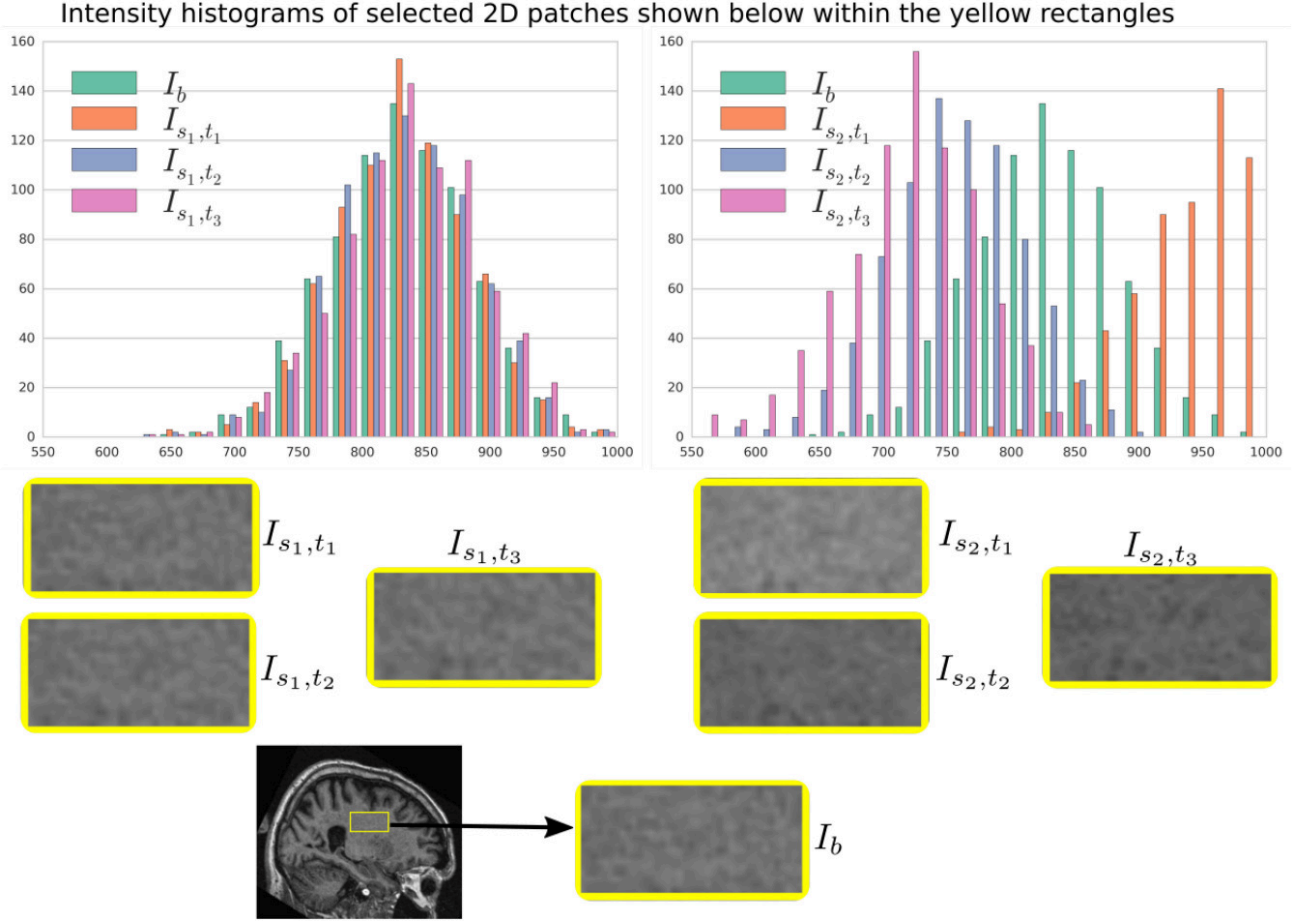
**Intensity histograms of selected patches of the images simulated in Figure 6**. When the simulated images are resampled from the same input baseline image *I*_*b*_, as expected, the histograms of the simulated images closely match with each other. However, when simulated images are resampled from other different images of the same patients, the histograms of these simulated images do not match closely. The longitudinal sequence of simulated images *I*_*s*_2_, *t*_1__, *I*_*s*_2_, *t*_2__, and *I*_*s*_2_, *t*_3__ has realistic variation in intensities as observed in the real sequences.

In Figure 6, a simulation example of two longitudinal sequences each having three new time-point images is shown. Both sequences were simulated by prescribing a smoothly varying atrophy pattern. The smoothly varying atrophy pattern prescribed in this example is more complex than the simple pattern used in the previous example. In brain parenchyma regions, it is the negative of the divergence of a stationary velocity field obtained by performing LCC log-Demons registration (Lorenzi et al., 2013) of the input baseline image with a follow-up image of the same subject. The first sequence consists of all the images whose intensities are resampled from the same input baseline image *I*_*b*_, while the second sequence consists of the images whose intensities are resampled from different real MRIs of the same subject. Thus, as shown in Figure 7, the first sequence does not have the realistic variation of intensities while the second sequence has the realistic variation of intensities. With this example, we also illustrated that we can generate multiple sequences of longitudinal images with same atrophy patterns but different variations of intensities.

**Figure 8:**
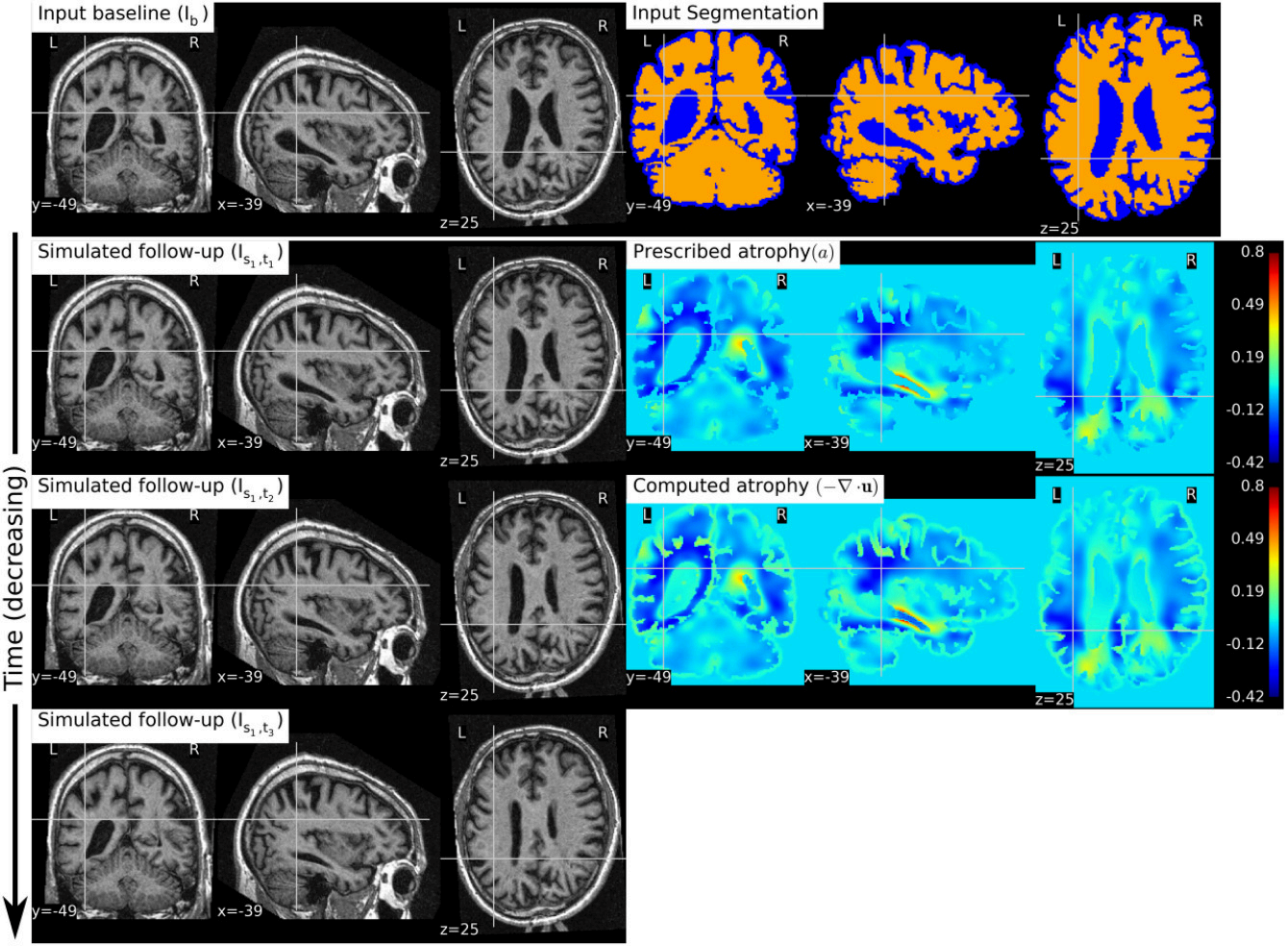
**The figure shows an example of simulating a longitudinal sequence with backward time-points**. The input baseline image *I*_*b*_ is the same one as used in Figure 6, and the prescribed atrophy map is the negative of the map used in Figure 6. In the figure, we can see the shrinkage of the ventricles and the growth of the brain parenchyma.

Figure 8 shows a simulation example where we prescribe growth instead of atrophy in the brain tissue. The prescribed atrophy in this case is the negative of the atrophy map prescribed in Figure 6. From the segmentation image shown in Figure 8, we can see that the ventricles were allowed to adapt the volume changes as required to compensate for the volume changes in the brain parenchyma. From the three simulated time-points, we can see that these ventricles are shrinking and the brain parenchyma regions are expanding. The example shows that Simul@trophy can be used to simulate images of not only future time-points, but also the past time-point images.

**Figure 9:**
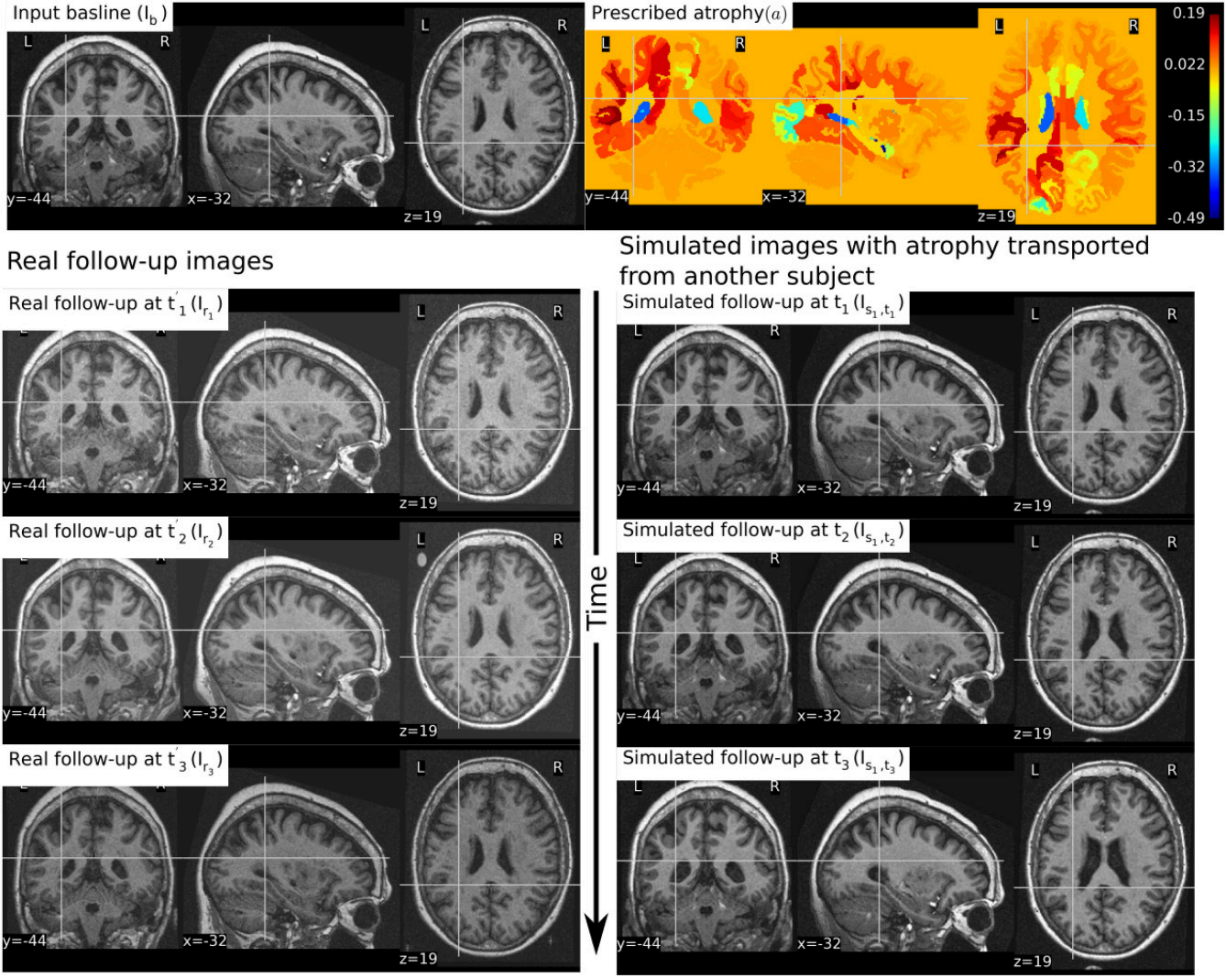
**The figure shows an example of simulating follow-up images of a normal subject with baseline image *I*_*b*_, where the prescribed atrophy pattern is adapted from an AD patient**. The prescribed atrophy is adapted from the atrophy estimated for the AD patient shown in Figure 6. Average values of the smoothly varying prescribed atrophy shown in Figure 6 is computed in all the ROIs. The ROIs are obtained from the FreeSurfer segmentation including all the white matter parcellations (Fischl et al., 2002). The simulated images on the right have bigger shrinkage of the brain parenchyma and bigger expansion of the ventricles than the real images on the left.

In Figure 9, we show an example where synthetic sequence of images is simulated by starting from a baseline image of a healthy subject. However, the prescribed atrophy is derived from an atrophy estimated from the AD patient used in Figure 6. The input baseline images of both the AD patient and the healthy subject were segmented using FreeSurfer (Fischl et al., 2002). In all the segmented regions including the white matter parcellations of the AD patient, the average values of the smoothly varying atrophy map were computed. These regional average values of the atrophy computed from the AD patient were then transported to the corresponding regions of the healthy subject. Thus, in Figure 9, we can see that the prescribed atrophy is region-wise uniform instead of smoothly varying. For comparison, the figure also shows three real time-point images of the healthy subject along with the three simulated time-point images with atrophy derived from the AD patient.

## 4. Simul@trophy: choices available and practical considerations

Simul@trophy is available as an open-source repository under git version control. Researchers can use it according to their needs, improve the presented model, and/or add new models of brain atrophy. It is based on two core components: (i) The Insight ToolKit (ITK) and (ii) PETSc Balay et al. (2013). All the input and output images of the brain deformation model shown in Figure 1 can be in any format that ITK supports. ITK has strongly promoted reproducible science in the medical imaging domain, and has been widely used in computational science applied to medical imaging (McCormick et al., 2014; Avants et al., 2015). Similarly, implementation of the model solver is based on open-source PETSc, a library based on C programming language. It has also been very widely used in a very diverse set of applications that also include the medical field. It is a very powerful library that supports wide range of iterative solvers and preconditioners for large systems of equations. The solvers implemented in PETSc can scale very well to large distributive computer systems.

Simul@trophy runs from command lines where the required inputs and optional choices are provided via command line arguments. The available command lines are detailed in Appendix in Supplementary Material. In this section, we illustrate some examples of how certain choices made during the simulation affect output results.

### 4.1. Impact of registration on simulated images

In Section 2.3, we explained that starting from an input baseline image of a subject, *I*_*b*_, we can generate two synthetic images:

$$I_{s_{1}}=\Phi_{\text{sim}}⋆I_{f}\text{ and }I_{s_{2}}=\left(\Phi_{\text{sim}}\circ \Phi_{\text{reg}}\right)⋆I_{f}$$

where Φ_sim_ is the deformation field obtained from the brain deformation model using *I*_*b*_ as the input baseline image, and Φ_reg_ is the deformation field obtained from the non-rigid registration between *I*_*b*_ and a real follow-up image *I*_*f*_. Perfect alignment of the two images with a non-rigid registration is possible only in the ideal case scenario. In such an ideal case, the simulated images *I*_*s*_1__ and *I*_*s*_2__ have identical shapes of the brain structures with the only differences lying in the intensity characteristics. In practice, this is almost never the case, and we present below an example of the impact of registration result on the simulated images.

Let us use the following short notations for various images described in this section.

- RB: Real baseline image: *I*_*b*_,
- RF: Real follow-up image: *I*_*f*_,
- RB_to_RF: Real baseline aligned to real follow-up: $\Phi_{\text{reg}}^{-1}⋆I_{b}$,
- SF_in_RB: Simulated follow-up image with intensity resampled from *I*_*b*_: Φ_*s*_⋆*I*_*b*_,
- SF_in_RF: Simulated follow-up image with intensity resampled from *I*_*f*_: (Φ_*s*_ ∘ Φ_reg_)⋆*I*_*f*_.

**Figure 10:**
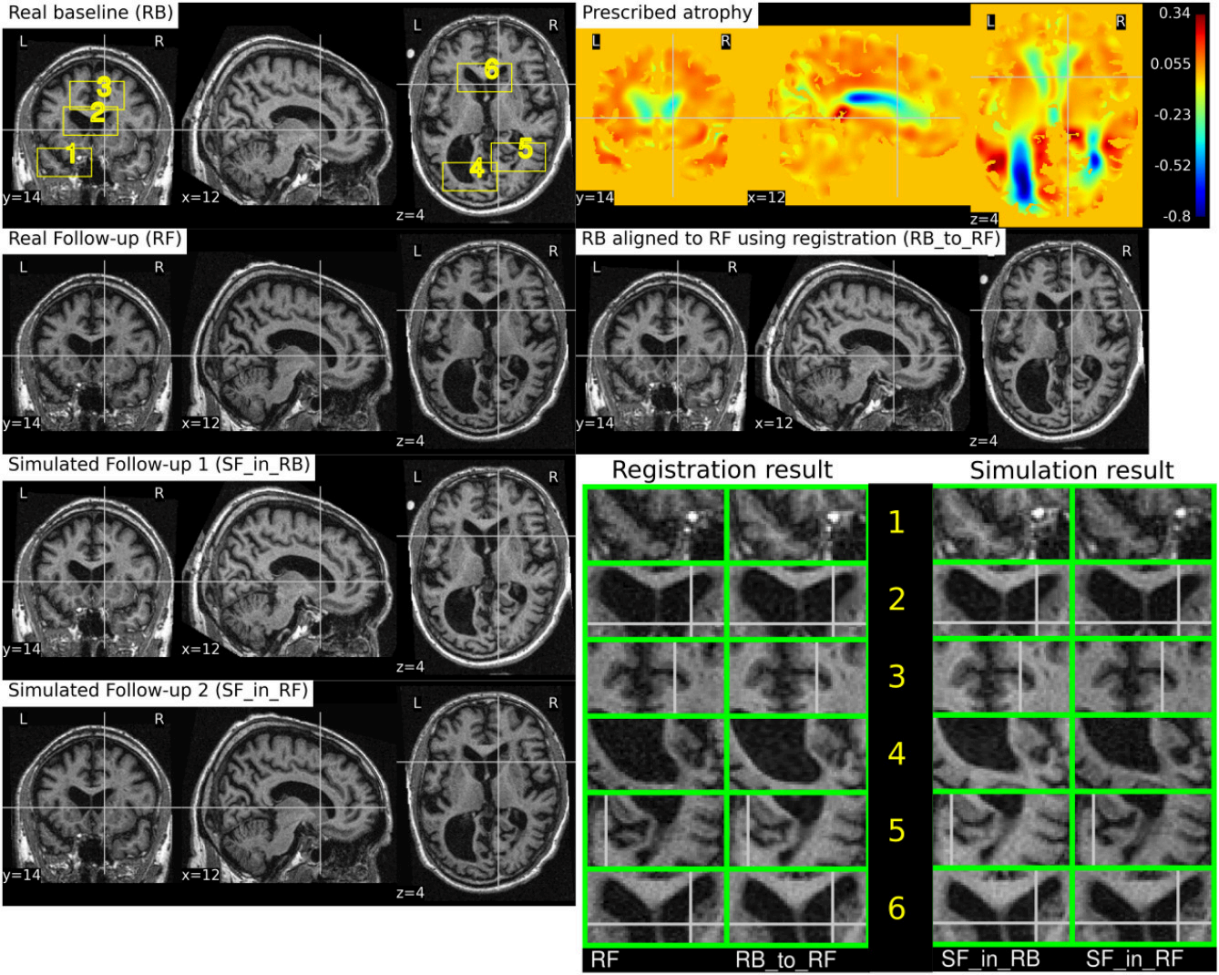
**RB and RF are non-rigidly registered and the transformation obtained from the registration is used to align RB to RF which is shown in the image RB_to_RF**. The figure also shows two simulated follow-up images SF_in_RB and SF_in_RF that are resampled from (RB) and (RF), respectively. We can see that in most regions of the brain, the two simulated images have almost identical morphological appearances. However, there are also regions such as 2 and 5, where the morphological appearances of the two simulated images are not identical. From the registration results for these regions 2 and 5 in the zoomed patches, we can see that the registration is also not accurate in those regions.

Figure 10 illustrates the impact of registration result Φ_reg_ on the simulation results. The figure shows both the registration and simulation results along with zoomed patches of RB, RB_to_RF, SF_in_RB, and SF_in_RF. As expected, SF_in_RB and SF_in_RF have different intensity characteristics coming from RB and RF, respectively. In the regions where registration is accurate, the two simulated images look almost identical except for the differences in the intensity characteristics. However, in the regions where registration is not accurate enough, SF_in_RB and SF_in_RF do not have identical shapes as expected. Thus, for the proposed method of using deformations obtained by registration for simulation, it might be preferable to use aggressive non-linear registrations with a much bigger weight given to similarity terms than the regularization terms.

### 4.2. Discretization scheme for the divergence computation

**Figure 11:**
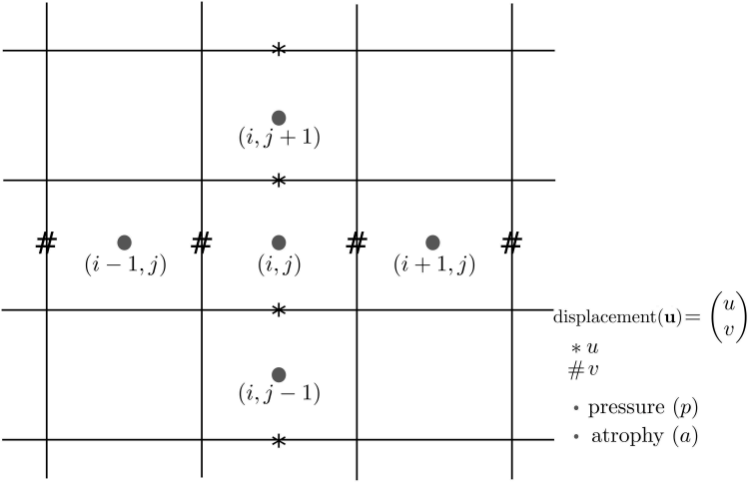
**Standard staggered grid discretization scheme that is used to solve the system of Equation (1)**. Displacement variables are at faces (edges in 2D) of the cells, while pressure and atrophy values are at centers of the cells.

In Khanal et al. (2016a), a standard staggered grid discretization was used for solving the system of Equation (1). The discretization scheme is shown in Figure 11 in 2D for illustration; explanation on 2D extends naturally to 3D. In the figure, we can see that the components of the displacement field variable **u** lie on cell faces and not at cell centers. However, all the input and output images for the model, including the output displacement field image, are standard images that have their values lying in cell centers or voxels. Our implementation of the solver internally creates the required staggered grid for the given input images. Once **u** is computed within the solver of system of Equation(1), its values at cell faces are interpolated to obtain the values at cell centers which are then assembled to send as output displacement field image. Within the solver, the numerical scheme used for the discretization of ∇ · **u** = −*a* is:

(2) $$\begin{array}{l} \frac{u_{i+1/2,j,k}-u_{i-1/2,j,k}}{h_{x}}+\frac{v_{i,j+1/2,k}-v_{i,j-1/2,k}}{h_{y}}+\\ \frac{w_{i,j,k+1/2}-w_{i,j,k-1/2}}{h_{z}}=a_{i,j,k} \end{array}$$

where,

$$u=\left(\begin{array}{c} u\\ v\\ w \end{array}\right).$$

Simul@trophy then provides output displacement field image with the values of **u** lying at cell centers or voxels by using linear interpolation as follows:

(3) $$\left(\begin{array}{c} u_{i,j,k}\\ v_{i,j,k}\\ w_{i,j,k} \end{array}\right)=\left(\begin{array}{c} \left(u_{i+1/2,j,k}+u_{i-1/2,j,k}\right)/2\\ \left(v_{i,j+1/2,k}+v_{i,j-1/2,k}\right)/2\\ \left(w_{i,j,k+1/2}+w_{i,j,k-1/2}\right)/2 \end{array}\right)$$

To compare divergence maps of this output field with the ones obtained from tools external of Simul@trophy, the only accessible values are the interpolated ones. ITK is widely used in registration based brain morphometry algorithms, but the default derivative computation of ITK has the following centered difference stencil:

(4) $$\frac{u_{i+1,j,k}-u_{i-1,j,k}}{2*h_{x}}+\frac{v_{i,j+1,k}-v_{i,j-1,k}}{2*h_{y}}+\frac{w_{i,j,k+1}-w_{i,j,k-1}}{2*h_{z}}=a_{i,j,k}$$

Replacing the components of **u** at cell centers from Equation 3, we get,

(5) $$\frac{u_{i+3/2,j,k}+u_{i+1/2,j,k}-(u_{i-1/2,j,k}+u_{i+3/2,j,k})}{4*h_{x}}+...=a_{i,j,k}$$

The scheme in Equation (5) does not match the one that was used internally by Simul@trophy shown in Equation (2). This results in discrepancy if we compare input prescribed atrophy maps against the externally computed divergence maps ∇ · **u**. Thus, in this work, we have added an implementation for the scheme in Equation (5) so that users can choose either of the two possible schemes of Equations (2, 5). The latter scheme is consistent with the divergence computed by the default derivative computation options of ITK. At each 3D cell, the scheme in Equation (2) involves 6 variables of the displacement field, while the scheme in Equation (5) involves 12 variables. In the rest of the paper, they will be referred to as 6-point and 12-point schemes, respectively.

**Figure 12:**
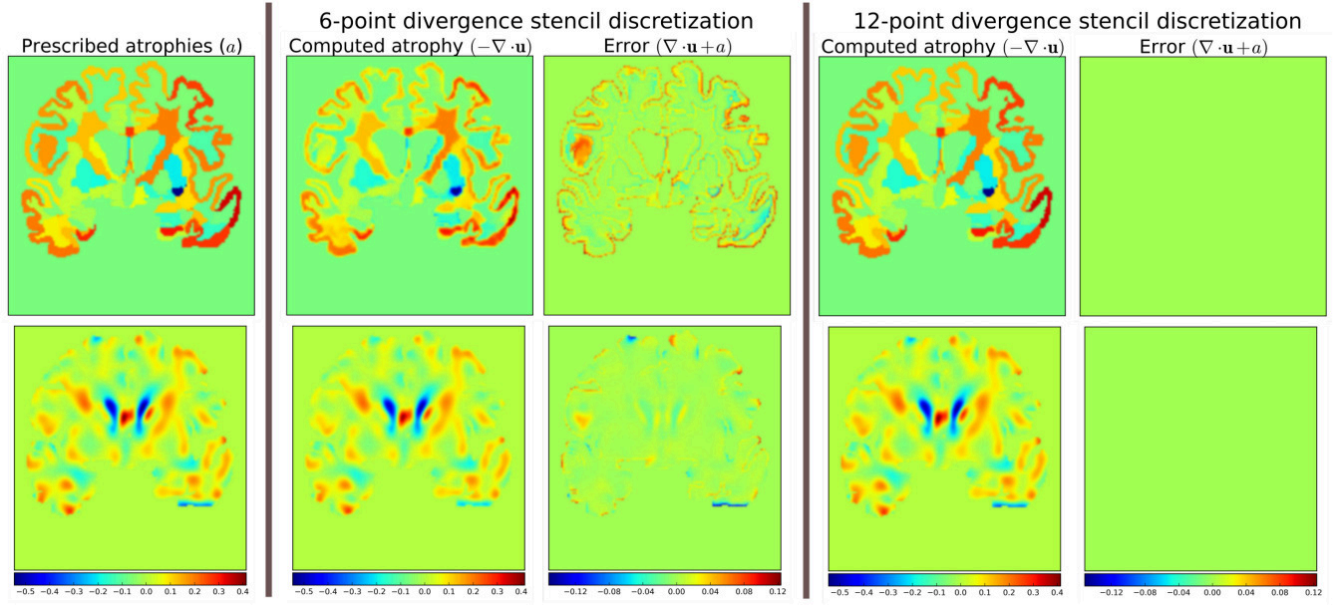
**Error due to non-consistent numerical schemes in Equations (2, 4, 5)**. ∇ · **u** shown in the figure are computed external of Simul@trophy by using the default ITK derivative computation scheme shown in Equation (4). When this divergence computation is consistent with the one used in Simul@trophy, we should obtain zero error with ∇ · **u** + *a* = 0. This is indeed the case, as seen on the right, when we use 12-point stencil of Equation (5). We see non-zero errors when using 6-point stencil from Equation (2) because this scheme and the default ITK scheme are not consistent. The figure shows that the error gets larger at areas where prescribed atrophy has discontinuous jumps.

Figure 12 shows the error in specified vs. obtained atrophy when using the two different numerical schemes. As expected, we can see that when a consistent numerical scheme is used, there is no difference between the specified and obtained atrophy. When the schemes are not consistent, the error is larger on the areas where the prescribed atrophy values change sharply.

If the simulated ground truth images using Simul@trophy are used for the evaluation of atrophy estimation algorithms, one must also be careful about the measure of volume change used in addition to the numerical scheme used. For instance, many TBM based brain morphometry algorithms use Jacobian determinants as a measure of volume change. To compute ground truth volume changes of the simulated images for the evaluation of such algorithms, users should compute Jacobian determinants using the same numerical scheme as used by the atrophy estimation algorithm being evaluated. For instance, if multiple time-steps was used in simulating the final image then the Jacobian must be computed at each individual step and properly accumulated to get the final volume change.

### 4.3. Implementation of image warping

When implementing an algorithm to warp an image with a given deformation field, it is more convenient to use the inverse of the deformation field. If Φ_*s*_ is the output deformation field obtained from the brain deformation model by using *I*_*b*_ as the input baseline image, Φ_*s*_ maps any point **x** in *I*_*b*_ to a point **y** in the simulated image *I*_*s*_ as follows:

$$y=\Phi_{s}(x).$$

However, **y** is not guaranteed to be a discrete voxel location. Since we do not know the intensity values of *I_s_ a priori* in the nearby discrete positions, the problem of interpolation is much more complex. Thus, we start from a discrete voxel location **y** in *I*_*s*_ where the value of intensity is to be found. Then, the corresponding position **x** in *I*_*b*_ can be obtained by using the inverse deformation field:

$$x=\Phi_{s}^{-1}(y).$$

If the transformed point **x** is not a discrete point, we can interpolate the intensities of *I*_*b*_ from neighboring discrete locations. Let us denote the interpolation by square brackets. Thus, *i* = *I*[**x**] describes a mapping of a point **x** to an intensity, *i*, of the MR image *I* at **x**. Using this notation, the intensity of the simulated image at any position **x** is given by:

$$I_{b}\left[\Phi_{s}^{-1}(x)\right].$$

The following option can be used to invert the deformation field:

--invert_field_to_warp #Invert  u;

      default:  do  not  invert

The implementation of the inversion is adapted from a fixed-point scheme implementation available in ITK (Luethi, 2010). By default, the simulator uses B-spline interpolation of order three to warp the input images.

### 4.4. Standalone utility tools and scripts for pre-processing and post-processing

There are some standalone tools and scripts available for various pre- and post-processing operations that are detailed in the documentation of the released software.

Some of these tools for pre-processing and post-processing operations are C++ executables based on ITK, while others are python scripts. In this work, all the input segmentation of the model were obtained by using FreeSurfer. As explained in Khanal et al. (2016a), these segmentation maps were processed to obtain in the format required by the model. Although the provided scripts are developed for FreeSurfer segmentation maps, they can be easily modified to adapt to other pre-processing tools. Finally, the registration and simulation deformations were composed using ComposeMultiTransform of Advanced Neuroimaging Tools (ANTs) (Avants et al., 2011).

The core component of Simul@trophy is the implementation of the brain deformation model. Resampling of the intensity is straightforward once the deformations from the model and from registration are available. The simulator is not dependent on any one particular registration algorithm. Although, we used LCC-LogDemons for illustrative purposes, this can be replaced with any other non-rigid registration algorihtms. Similarly pre-processing is also independent of Simul@trophy. We used FreeSurfer in the simulation examples shown in this work, but any other skull stripping and segmentation algorithms can be used. Simul@trophy provides some example scripts and some utility scripts, which could be modified when using other tools for the pre-processing step.

## 5. Discussion

In Khanal et al. (2016a), we presented a method to generate a subject-specific atrophy pattern by first measuring the atrophy from the available time-points, and then simulating a new time-point by prescribing the measured atrophy. In Khanal et al. (2016b), we extended the method to interpolate an unavailable intermediate time-point MRI. In this work, we added realistic variation in the intensity of the synthetic images. This fills an important gap in the existing literature to simulate atrophy in longitudinal images with realistic intensity variation without explicitly modeling the noise and acquisition artifacts. The simulation examples were shown using three types of atrophy patterns: (i) very simple uniform volume changes in small number of regions, (ii) uniform atrophy in large number of regions, and (iii) smoothly varying atrophy patterns.

For each subject, we could generate large number of synthetic images by perturbing these atrophy patterns in different ways. Even with the same atrophy pattern, we can generate multiple sets of longitudinal sequences of varying intensity characteristics using the approach illustrated in Figure 4. Thus, by changing the atrophy patterns and the image intensities, Simul@trophy could be used to generate a database of very large number of simulated images. Such a database might be useful for training of machine learning algorithms.

In Figure 6, smoothly varying atrophy pattern was prescribed by taking the negative of the divergence of a stationary velocity field obtained by registering the input baseline image with a follow-up image of the same subject. The objective of the experiment was to illustrate the ability of Simul@trophy to simulate smoothly varying patterns of atrophy in addition to the piecewise continuous atrophy maps. Registration was taken just as a means of getting a realistic smoothly varying atrophy maps; it is worth mentioning that simulating the deformation to be close to the deformation obtained from the registration algorithm was not the objective of this experiment. This is because the actual deformation field depends on the regularization used in the registration algorithm which does not necessarily follow the modeling assumptions used by Simul@trophy.

Although, the proposed method of resampling intensity from an image different from the input image provides more realistic variations, there are nevertheless certain issues one needs to be aware of. Since the simulated image has its intensities interpolated from another image, it can slightly reduce the noise variance. A neighborhood with expansion in the simulated image have intensities with slightly different linear combinations of intensities coming from a smaller set of voxels in the input image. Thus, the simulated image would have a smoother autocorrelation in the neighborhood compared to an equivalent real image. The fact that the simulated image has undergone interpolation and draws intensities from a limited set of raw voxels means that it is inherently smoother than the real scans. Finally, the usual spatial patterns of artifacts on real scans might not be exactly reproduced when warping real images. Any application using the simulated sets of images with the proposed approach should be aware of and ideally take into account these issues when interpreting results.

Use of repeat baseline scans to obtain intensity variation in the simulated images provides a very simple approach without using explicit noise and artifact models. One limitation with this is that the repeat baseline scans are not always available. When repeat scans are not available, we have proposed to use images at other time-points of the same subject, which requires performing non-linear registration. However, none of the non-linear registration methods are perfect and therefore the inaccuracies in registration affect the simulation results. This issue was discussed with illustrative examples in Section 4.1.

Simul@trophy can be used in evaluating atrophy estimation algorithms in similar ways as done by Pieperhoff et al. (2008), Camara et al. (2008), and Sharma et al. (2010). Since the proposed approach to simulate images may need deformations estimated from image registration, the use of these simulated images for the evaluation of some registration algorithms can bring an issue of circularity. This limitation adds to another limitation present in all publications related to atrophy simulation that we are aware of: namely, the models used in simulating images could favor certain kinds of registration algorithms over others. Although the ground truth atrophy can be measured from the combined deformation fields, the users must be aware of both limitations when they use Simul@trophy for the evaluation of registration algorithms.

The ability to prescribe atrophy at any time point allows the user to introduce volume changes at different regions of the brain at different times. Thus, another interesting application of the simulator is to train and/or validate disease progression models such as the models proposed in Chen et al. (2012), Fonteijn et al. (2012), Jedynak et al. (2012), Dukart et al. (2013), and Schmidt-Richberg et al. (2016). Having a database of longitudinal MRIs with known spatio-temporal distribution of atrophy can be useful to validate such algorithms. Furthermore, since the algorithms use a data driven approach, the simulator could be useful to train or fine-tune such models.

Another possible application is in filling up unavailable time-point MRIs of some of the subjects, when performing group-wise longitudinal analysis. In such studies, usually the available time-point images of each subject are used to estimate subject-specific volume changes. These subject-specific measurements are then used to perform group-wise statistics to check whether there are significant differences amongst different groups in some particular regions of the brain. Databases used in such analyses, might not always have all the required time-point images for all the subjects. This could lead to bias if all the subjects are not aligned properly in the temporal dimension of disease progression. Simulating new time-point images for some subjects and using them in the analysis might allow evaluating the impact of such mis-alignments.

Simul@trophy could also be used in studying the role of morphology and intensity on atrophy estimation algorithms, and in machine learning based AD classification algorithms. Simul@trophy enables to perform such studies as it allows creating a large number of images by simulating atrophy patterns commonly observed in AD patients but with intensities taken from normal subjects and vice versa.

We hope to promote two directions of research in the community with open-source release of Simul@trophy. *First*, the public availability of Simul@trophy enables researchers to build their own simulated databases as needed. This might also hopefully lead to a large public database of ground truth simulated images, that could be used for benchmarking and evaluation of various image based morphometry tools. *Second*, we hope that Simul@trophy allows other researchers to build upon the biophysical model we presented in Khanal et al. (2016a), and investigate further, providing more accurate models of brain atrophy.

Finally, Simul@trophy is general enough to be used for other imaging modalities such as CT scans. It could also be used with images of any other organs, where one requires simulating specified volume changes. In this case, the pre-processing should be changed accordingly to generate a segmentation image and atrophy maps. Thus, once the software is public, other researchers might find it useful in applications that we have not foreseen yet.

## 6. Conclusions

We proposed a simulation framework that can generate realistic longitudinal MRIs with specified volume changes. The framework allows generating large number of subject-specific multiple time-point images based on a biophysical model of brain deformation due to atrophy. We developed an open-source software Simul@trophy to implement the proposed framework. The core part of Simul@trophy is the implementation of our brain deformation model presented in Khanal et al. (2016a). Simul@trophy is based on widely used state of the art libraries PETSc (for solving large systems of equations) and ITK (for medical image processing). Since the software is publicly available in an open-source repository, we hope that researchers can use it to create databases of ground truth images. The framework could be used to generate a common public database, which in turn could be used to validate and evaluate a large number of available atrophy estimation algorithms. Similarly, these databases could be valuable for data driven disease progression models including machine learning algorithms. Validation and training of the models that study temporal relationships, ordering, and co-evolution of atrophy in different structures of the brain could be another interesting application.

## Author contributions

BK has worked on the design and implementation of Simul@trophy. NA and XP have supervised him in the design of the experiments and in the methods presented in the paper. BK has written the manuscript with multiple iterations of suggestions and corrections from NA and XP.

## Funding

Part of this work was funded by the European Research Council through the ERC Advanced Grant MedYMA 2011-291080.

### Conflict of interest statement

The authors declare that the research was conducted in the absence of any commercial or financial relationships that could be construed as a potential conflict of interest.

## Footnotes

^1^Available at: https://inria-asclepios.github.io/simul-atrophy/.

^2^The simulation results are made available at http://neurovault.org/collections/AUKWWYBC/ (Gorgolewski et al., 2015).
