# Supplementary material for: Simulating Longitudinal Brain MRIs with Known Volume Changes and Realistic Variations in Image Intensity
Source: Front Neurosci. 2017 Mar 22;11:132. doi: 10.3389/fnins.2017.00132 (PMC5360759; doi:10.3389/fnins.2017.00132)
Supplement: Supplementary file 1 [file DataSheet1.pdf]

APPENDIX

Running `Simul@trophy` from command lines

Once the pre-processing steps described in Section 2.1 are performed and the desired atrophy map is generated, these images can be used as input to the model by providing the following command line arguments:

```
-atrophyFile            #Input atrophy map
-maskFile               #Input segmentation file
-imageFile              #Input image file
```

If the model parameters μ and λ have uniform values in `Label1` and `Label2`, they can be provided as an argument to the option `-parameters`. On the other hand, if they need to have different values in different parts of the brain, one needs to provide them as images similar to other input images as shown below:

```
-parameters             #μ,λ in Region1, Region2. Format: μ1,μ2,λ1,λ2
-muFile                 #Ignore μ from -parameters, use this image
-lambdaFile             #Ignore λ from -parameters, use this image
--useTensorLambda       #λ given as DTI; default is scalar image
```

Some of the important options available are:

```
-boundary_condition     #dirichlet_at_walls or dirichlet_at_skull
--div12pt_stencil       #Use 12-point scheme; default: 6-point scheme
--relax_ic_in_csf       #Region1: ∇·u+kp=0; default is ∇·u=−a
-relax_ic_coeff         #Value of k
-numOfTimeSteps         #Number of time-steps to solve for
```

To solve the system of Eqs. (1), the argument to `-boundary_condition` should be `dirichlet_at_skull` and `--relax_ic_in_csf` must be provided. Using `dirichlet_at_walls` instead of `dirichlet_at_skull` will consider regions with `label0` in the same way as the regions with `label2`, and sets the Dirichlet boundary conditions only at the image borders.

If `-numofTimeSteps` is greater than one, the simulator provides an output displacement field obtained by composing output displacement fields of each time-steps. For any time-step `n` < `numOfTimeSteps`, it also provides output synthetic image by warping the input image with the displacement field obtained by composing output displacement fields from time-step 1 to `n`. In addition to these outputs, if desired, some other extra outputs can be generated as shown below:

```
-resPath                   #Result path to store all the results
-resultsFilenamesPrefix    #Prefix to be provided to all the images
--writePressure            #Write p as image to disk.
--writeForce               #Write (μ+λ)∇a as image to disk.
--writeResidual            #Write solver residual as image to disk.
```
